# On the Medical Properties of the Natural Order Ranunculaceæ; and More Particularly on the Uses of Sabadilla Seeds, Delphinium Staphisagria, and Aconitum Napellus, and Their Alcaloids, Veratria, Sabadilline, Delphinia, and Aconitine

**Published:** 1836-10

**Authors:** 


ART. VI.—*On the Medical Properties of the Natural Order Ranunculaceæ; and more particularly on the uses of Sabadilla Seeds, Delphinium staphisagria, and Aconitum napellus, and their Alcaloids, Veratria, Sabadilline, Delphinia, and Aconitine.* By A. TURNBULL, M.D.—*London,* 1835. 12mo. pp. 171.

IF one quarter of what is contained in this book be true, (and surely that is not an unreasonable requisition,) Dr. Turnbull's alkaloids are sufficient to change the face, not only of medicine, but of society. Here and there an eccentric annuitant might be found who would allow himself to be cut off by hypertrophy of the heart at fifty or sixty; but, the majority would keep a box of veratria ointment on their dressing tables, and live to eighty-six or ninety, to the dismay of the Atlas and the Globe. Dr. Turnbull, indeed, very modestly says

"The author would caution the profession against expecting too much from the employment of these remedies. In some cases they have given only a temporary relief, whilst in others they have had no effect: but generally speaking he has found them of much more advantage in the treatment of a very distressing class of affections, than any means hitherto discovered, and on this account he would recommend their use." (*Preface,* p. 6.)

This is just as if a guide were to introduce a traveller to the falls of Niagara with a caution not to be surprised. How can we expect too much from veratria, which, of nine cases of affections of the heart, is said to have cured or relieved all; of thirteen cases of neuralgia, to have cured twelve; of nine cases of rheumatism, to have cured eight, and almost the remaining one? The heart affections, moreover, were evidently cases of organic disease, though the author, with great humility, generally refrains from saying so: e. g.

"CASE IV. Mr. J., a clergyman, fifty years of age, has been affected with severe palpitation for the last seven years, accompanied by quickness and irregularity of the pulse, difficulty of breathing, loss of voice, cough, expectoration, and a distressing sense of anxiety: has sometimes been seized in the pulpit with giddiness, succeeded by throbbing in the neck and confusion of intellect, and these symptoms have occasionally gone on to such an extent as to oblige him to desist from his duties for two or three months at a time: his bowels are regular, and his digestion generally good.

"He was ordered to take small doses of tartar emetic, and to have a blister applied over the chest; and this treatment was pursued with considerable advantage for the time, but when it was remitted he soon returned to the same state as before. As this seemed a fair case upon which to make trial of the veratria, it was ordered to be rubbed on in the manner already described. By making use of the frictions once every night he became gradually better, and at the end of a week considered himself quite well; he was advised, however, to continue the ointment for a little longer, and then to leave it off by degrees: this was accordingly done about a year and a half ago, and he has remained ever since in excellent health, free from his old complaints, and able for the discharge of the functions of his office." (P. 39.)

Every case of neuralgia was perfectly cured, except one, and even of that the history concludes, in the text, with the words "leaving no trace of the affection behind, neither has any renewal of it taken place." (p. 70.) The note, however, a traitor to the text, says, "In this instance the veratria has completely failed in giving permanent relief," &c.

Dr. Turnbull also recommends veratria in gout, dropsy, and paralysis; but, in the last disease, he says, that tincture of sabadilla deserves the preference.

Delphinia, like veratria, is chiefly employed externally, and in the same diseases; and, as it can be applied to mucous membranes without inflaming them, is in some cases preferable for that reason; and, in others, for its property of exciting the circulation of the part to which it is applied. One case only is given in which it was employed, a facial neuralgia, which was relieved by veratria, but perfectly cured by delphinia.

Aconitine is employed externally, in the form of ointment, like veratria. A case is given of agonising neuralgia of the fingers, which, after having long resisted a variety of remedies administered by Dr. Elliotson, was cured by Dr. Turnbull with the tincture of the root of aconite and ointment of aconitine.

There is an appendix containing several cases successfully treated by the veratria ointment, and communicated to the author by Drs. Macgowan and Hood and Messrs. Holme, Spence, and Porter.

On the whole, we must come to this impartial but perplexing conclusion: if Dr. Turnbull's cases are fairly told, veratria (for the other alkaloids are of very secondary importance,) is the greatest discovery of the age, and Dr. Turnbull is the most skilful or most fortunate of practitioners; if, on the other hand, we are to trust to our own experience, (for we too have tried the alkaloids,) and to the experience of friends who have tried them much more extensively, the utmost that we can say in their favour is, that, like many of the medicines in daily use, they are at most if not of doubtful, at least of uncertain, efficacy. Sometimes they have done good, particularly in neuralgia; frequently they have proved of none effect: in no case that has come under our own immediate observation could we feel authorised, by the laws of legitimate induction, to pronounce more highly of their merits than of twenty other remedies, in the hands of every one, and long prescribed in the same diseases. Still we are far from saying that these alkaloids are valueless, or that they do not deserve the careful attention of the profession; neither do we regard Dr. Turnbull's efforts or Dr. Turnbull's book as claiming from his brethren unqualified contempt or censure. On the contrary, we believe in the efficacy of the drugs, and we are not unthankful to the author, who has taken much pains to make them known; but we condemn most unreservedly the careless, hasty, and unphilosophical manner in which the investigation has been conducted, and the blind and headlong zeal which has done its utmost to ruin the cause it would recommend, in the minds of all sober thinkers.

If veratria can stand the ordeal of such injurious partisanship,—at least in these our days,—it must indeed be a remedy of no mean virtue. We conclude by recommending such of our readers as have not yet made trial of these preparations, to do so, but with a more sedate mind and in a more critical spirit than the present publication evinces its author to have done; and to give the results as judges, not as advocates, careless for the name or fame of veratria, and solicitous only concerning truth.

